# Tissue and Serum Trace Elements Concentration among Colorectal Patients: A Systematic Review of Case-Control Studies

**Published:** 2019-04

**Authors:** Azmawati Mohammed NAWI, Siok-Fong CHIN, Shamsul AZHAR SHAH, Rahman JAMAL

**Affiliations:** 1. Universiti Kebangsaan Malaysia Medical Molecular Biology Institute (UMBI), Universiti Kebangsaan Malaysia Medical Center, Kuala Lumpur, Malaysia; 2. Department of Community Health, Faculty of Medicine, Universiti Kebangsaan Malaysia, Kuala Lumpur, Malaysia

**Keywords:** Systematic, Review, Trace element, Colorectal cancer (CRC), Case-control

## Abstract

**Background:**

Trace elements play a pivotal role in Colorectal Cancer (CRC) inhibition and development process. This systematic review provides the basic comparison of case-control studies focusing on concentration of trace elements between those with CRC and controls

**Methods:**

The systematic review searched through two databases of Medline and Cochrane up to 24^th^ June 2017. The search strategy focused on Population, Intervention, Comparison, and Outcomes (PICO). We searched the role of trace elements in cancer and focusing on case-control studies in CRC to obtain an insight into the differences in trace element concentrations between those with and without cancer.

**Results:**

The serum concentrations of Ca, Cu, Mg, Mn, Se, Si, and Zn were lower in CRC patients but for Co and S the levels were higher in CRC patients. The concentrations of Cd, Cr, Cu, Mg, Mn, Pb, and Zn were increased in patients with metastasis, but not in Se. As for colon tissue specimens, inconsistent levels were reported between studies, notably in Cu, Se, and Zn. No changes were reported for B and Ca levels. Most of the trace elements in the tissue specimens showed higher concentrations of Cr, Fe, K, Mg, P, Rb, S, and Si compared to Br.

**Conclusion:**

With the growing interest to understand the link between trace elements in carcinogenesis and the possible interactions, multi assessment analysis of a larger cohort of samples is necessary.

## Introduction

Colorectal cancer (CRC) incidence rates and trends contributed to the significant variation that occurs between geographical regions and across nations. Globally, CRC is the third most common cancer in adult males and second among females (1, 2). In Asia, the incidence and mortality rates are gradually increasing with ethnicity emerging as one of the key risk factors whereas the incidence is becoming analogous to the Western countries (3). The incidence has increased from 1987 to 2002 for both males and females in every cancer registry especially in Japan. However, some countries such as India and Thailand have shown relatively low incidence rates (4). In Singapore, the incidence rate was 26.7%–38.2% in 2010–2014 among female and male populations (5) and it was 21.3% among Malaysian from 2008–2013 with the Chinese showing the highest incidence (6). Lifestyle-related factors such as the Western diet (7), alcohol intake (8), smoking habit (9), obesity (10) and sedentary activities (11) are strongly associated with a higher risk of CRC, besides the genetic factors (12) and a history of polyps or inflammatory bowel disease (13). Dietary intake appears as the main source of exogenous trace elements present in the human body (14). Many of the metabolic and physiological processes in our body require trace elements (15). Major elements account for about 96% of the total elements in the human body while the remaining 4% are trace elements (16). Despite their presence in smaller amounts, these trace elements exert tremendous influence on many body functions including mediating vital biochemical reactions by acting as cofactors for many enzymes, stabilizing structures of enzymes and proteins, controlling important biological processes by binding to molecules on the receptor site of cell membrane, and alternating the structure of membrane to prevent entry of specific molecules into the cell (15, 16). The imbalances in various trace elements have been observed in many diseases including heart disease, autoimmune disease, cancer, renal failure and neurological disorders (17, 18).

The accumulation or deficiency of these elements may stimulate an alternative pathway towards the development of CRC. Several trace elements are considered as chemical carcinogens that may change the biological processes and induce carcinogenic effects (19, 20). These genotoxic carcinogens are capable of altering the genetic makeup of target cells (19) causing multiple mutations in the critical genes in the human body which may lead to accumulation of irreversible DNA damage and ultimately cancer development (20). Apart from its genotoxic role, trace elements have also been reported as non-genotoxic carcinogens (21) that act via secondary mechanisms such as tumor promoters (22), endocrine-modifiers (23), receptor mediators (24), immunosuppressants (25), or inducers of tissue-specific toxicity and inflammatory responses (26). These non-genotoxic mechanisms vary diversely and are complex, hence they remain challenging to comprehend in terms of their full carcinogenic potential. The delicate involvement of trace metals in the multifaceted processes of cancer growth and inhibition has launched the quest to decipher their essentiality and toxic effects on human health (20).

Our immune system is the most common and natural defense mechanism against cancer that orchestrates the function of phagocytosis, complement system, lysosomes, immunoglobulins and interferons upon abnormal cell transformation. Reinforcement on this defense system in response to malignancy by the presence of trace elements such as zinc (Zn), selenium (Se) and manganese (Mn) has been reported previously (27–29). The participation of these elements in the immunological processes against cancer can be likened to other body functions (30) and thus, the balance between essential and toxic elements is thought to be crucial in impeding the development of cancer.

We performed a systematic review on the role of trace elements in cancer and focusing on case-control studies in CRC to obtain an insight into the differences in trace element concentrations between those with and without cancer.

## Materials and Methods

We adopted the process of conducting a systematic review as outlined by the Cochrane Collaboration (31) and the reporting guidelines for Meta-Analysis of Observational Studies in Epidemiology (32).

### Comparison and Outcomes Search strategy

Two databases were searched, i.e. Medline and Cochrane Databases up to 24^th^ June 2017, and our search was limited to English-language publications. The search was performed based on the PICO: Population (CRC), Intervention (not related with current search), Comparison (Non-CRC, controls or healthy) and Outcome (trace elements level). The following search strategy was applied: colorectal adj1(carcinoma* or tumo? r* or cancer* or neoplasm*).tw OR colon* adj1(cancer* or neoplasm*).tw OR cancer adj3(colon*).tw OR sigmoid* adj1(neoplasm* or cancer*).tw OR cancer adj3(sigmoid*).tw OR sigmoid colon adj1(neoplasm* or cancer*).tw OR rectal adj1(tumo?r* or cancer* or neoplasm*).tw OR cancer adj3 (rectum*).tw OR rectum adj1(cancer* or neoplasm*).tw AND Biometal*.tw OR trace adj1(element*).tw OR micronutrient*.tw

### Inclusion and exclusion criteria

The title and abstract of articles were first screened from the initial search, followed by a review of the full-text articles of potentially eligible studies for further selection. The list of citations from the shortlisted articles was scanned for potential missing studies. The retrieved list of articles was carefully examined to exclude possible duplicates or overlapping information. Two reviewers assessed the full-text article independently by using a specific data extraction form. Studies were eligible for inclusion if trace element concentrations were measured in both colorectal cancer patients and healthy controls. Only studies that measured trace element status in colon tissue or serum with more than one trace element were included. In addition, only articles that reported a case-control study design were reviewed in their full text. Disagreements arose over 6% of the articles (κ=0.89) and this was resolved by discussion among reviewers and a third party.

### Data extracted

A standardized data extraction tool and a study quality grading instrument were developed. One reviewer extracted the data while the other reviewer checked the information for accuracy. Data extracted from each study include the information on the first author, year of publication, country, patient characteristics (including sample size, gender and mean age) and concentration of trace elements. Study quality was based on our own tool mainly adopted from the Grading of Recommendations Assessment, Development and Evaluation (GRADE) risk of bias assessment for observational studies (33). The study biases, including methodological domains of participant selection, result measurement, exposure measurement, control for confounding and appropriate analysis were measured by a simple checklist with a key domain. Low, moderate or high risk of bias was evaluated for each study included in this review.

## Results

**Fig. 1:**
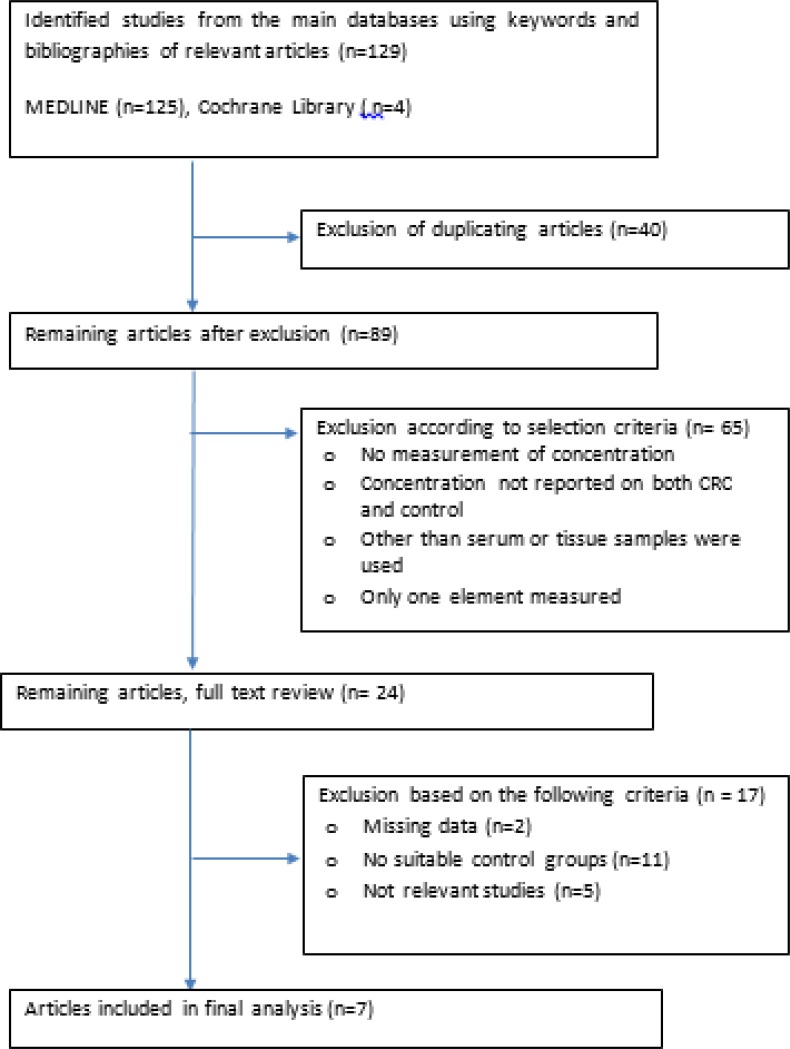
A systematic review study flow on trace elements determination in CRC patients

Out of the 129 publications identified, seven articles were relevant and one additional article was identified through the citation list of published work (Fig. 1). Four articles were related to serum concentrations of the trace elements while another four related to concentrations in the colon tissues. Some of the serum trace element levels were significantly different between colorectal and non-colorectal cancer patients, such as Co, and some elements notably Cd and Zn were even higher in the metastatic tumors (Table 1). The levels of trace elements in tissues were dissimilar, however, only Co and Mg concentrations were significantly different as compared to controls. The quality of articles regarding the risk of bias was low (Table 2).

**Table 1:** Serum trace elements concentration between colorectal patients and controls

***Study (yr)***	***(Gupta & Shukla, 1993)***		***(Shenberg et al., 1995)***		***(Milde et al., 2001)***		***(Emre et al., 2013)***	
***Place***	***India***		***Belgium***		***Czach Republic***		***Turkey***	
***Sample***	***Healthy (n=30)***	***Tumour (n=30)***	***Healthy (n=10)***	***Tumour (n=11)***	***Healthy (n=10)***	***Tumour (n=20)***	***Healthy (n=29)***	***Metastatic Tumour (n=40)***
Technique	AAS		INAA & PIXE		AAS		AAS	
Ca	NA	NA	0.936± 0.195	0.935± 0.106	NA	NA	NA	NA
Cd	NA	NA	NA	NA	NA	NA	0.0027± 0.0003	0.1832± 0.1441^*^
Co	98.84 ±24.31	165.99± 33.90^*^	NA	NA	NA	NA	NA	NA
Cr	NA	NA	NA	NA	NA	NA	0.0061± 0.0016	0.0325± 0.0080*
Cu	NA	NA	NA	NA	1.2± 0.1	0.90± 0.20	0.151± 0.0245	0.241± 0.1004^*^
Li	NA	NA	1.270 ±0.090	1.212± 0.105	NA	NA	NA	NA
Mg	NA	NA	NA	NA	20.9 ±0.8	18.23± 2.93	10.81 ±1.4231	32.838± 11.8095^*^
Mn	NA	NA	1.042 ±0.183	0.875± 0.180	NA	NA	0.2862± 0.0486	0.3326 ±0.2063^*^
Pb	NA	NA	NA	NA	NA	NA	0.0268± 0.0077	0.1788± 0.0705^*^
Se	NA	NA	NA	NA	74.5± 2.5	0.041± 0.009	0.1138± 0.0294	0.05534 ±0.0233^*^
S	NA	NA	0.796± 0.103	0.807± 0.161	NA	NA	NA	NA
Si	NA	NA	1.261± 0.190	1.156± 0.096	NA	NA	NA	NA
Zn	115.08±16.1^a^	93.21±13.43^a*^	NA	NA	1.4± 0.1	1.03± 0.57	2.488± 0.332	5.134± 1.8658^*^

INAA Instrumental Neutron Activation Analysis, PIXE Particle/proton Induced X-ray emission, AAS Atomic absorption spec-trometry, concentration in ngg-1,

* statistically significant (*P*<0.05),

NA: not available

**Table 2:** Trace elements concentration in colon tissues

***Study(year)***	***(Gupta & Shukla, 1993)^a^***		***(Milde et al., 2001)***		***(Magalhães et al., 2010)^c^***		***(Rinaldia et al., 2015)^b^***	
***Place***	***India***		***Czach Republic***		***Germany & Portugal***		***France***	
***Sample***	***Healthy (n=30)***	***Tumour (n=30)***	***Healthy (n=10)***	***Tumour (n=20)***	***Healthy (n=15)***	***Tumour (n=15)***	***Healthy (n=28)***	***Tumour (n=76)***
Technique	AAS		AAS		TXRF		ICP-AES	
Weight	dry		dry		wet		wet	
B	NA	NA	NA	NA		NA	0.4 (1.5)	0.4(4.1)
Br	NA	NA	NA	NA	↑		NA	NA
Ca	NA	NA	NA	NA		unchanged	NA	NA
Co	1.79±0.57	2.78 ±0.84*	NA	NA		NA	NA	NA
Cr	NA	NA	NA	NA		NA	5.7(3.9)	7.9 (8.8)
Cu	NA	NA	15.9± 0.6	6.08 ±3.78		↑	NA	NA
K	NA	NA	NA	NA		↑	NA	NA
Mg	NA	NA	708± 21	753.59± 310.54		NA	103.3(37.1)	147.0(52.2)*
Mo	NA	NA	NA	NA		NA	NA	NA
P	NA	NA	NA	NA		NA	NA	NA
Rb	NA	NA	NA	NA		NA	NA	NA
Se	NA	NA	0.86± 0.07	1.17 ±0.73	↑		NA	NA
S	NA	NA	NA	NA		NA	NA	NA
Si	NA	NA	NA	NA	NA		11.2 (11.8)	22.3 (29.9)
Zn	27.16± 9.11	18.98± 6.41*	178± 6	69.20±21.03	↑		15.7(12.9)	19.7(12.5)

AAS Atomic absorption spectrometry, TXRF Total reflection x-ray fluorescence, ICP-AES Inductively coupled plasma atomic emission spectrometry

a Concentration in μg g-1,

b Results presented median (IQR),

c not stated the value,

* statistically significant (*P*<0.05),

NA: not available,

↑: increased

### Population studied

The sample size of colorectal patients analyzed within each of the selected studies ranged from 11 to 76. The average age ranged from 45 to 85 yr old and the majority of the patients were female (68%). Most of the cases were of Dukes’ B stage and most of the studies involved hospital-based patients. The main exclusion criteria for case and control are patients with history of polyps, inflammatory bowel disease, and concurrent cancer.

### Trace elements concentration

Atomic Absorption Spectrometry (AAS) application was the most popular acquisition used in these studies, accounting up to 62%. Table 3 summarizes the trace element concentration of serum and colon tissue specimens. The serum concentrations of Ca, Cu, Mg, Mn, Se, Si, and Zn were generally lower in the colorectal cancer patients but higher in Co and S. Nonetheless, the concentration of the listed elements increased in the majority of cases of metastatic disease, except for Se.

**Table 3:** Findings summary of trace elements concentration in serum and tissue

***Elements concentrations***	***Blood serum***		***Tissue***
	***Tumour***	***Metastatic Tumour***	***Tumour vs healthy***
B	NA	NA	unchanged
Br	NA	NA	↓
Ca	Slight ↓	NA	unchanged
Cd	NA	↑	NA
Co	↑	NA	NA
Cr	NA	↑	↑
Cu	↓	↑	↑/↓
Fe	NA	NA	↑
K	NA	NA	↑
Mg	↓	↑	↑
Mn	↓	↑	NA
P	NA	NA	↑
Pb	NA	↑	NA
Rb	NA	NA	↑
Se	↓	↓	↑/↓
S	↑	NA	↑
Si	↓	NA	↑
Zn	↓	↑	↑/↓

NA: not available,

↑: increased,

↓: decreased

As for the concentration in colonic tissue specimens, inconsistent results were reported between studies namely on Cu, Se and Zn elements whereas B and Ca did not differ between colorectal and non-colorectal patients. Most of the trace elements in tissue specimens showed higher concentrations including Cr, Fe, K, Mg, P, Rb, S and Si.

## Discussion

The knowledge gap that we aim to fill from this systematic review is to address the possible association between trace elements and colorectal cancer. From the extracted data, inconsistent concentrations of trace elements reported for the sera and colon tissues were noted between the studies. The variation is expected as different instruments were used for measurement and acquisition, hence the inevitably varied detection limits and readings. This variation is more pronounced in studies using tissue specimens compared to those which measured the serum and thus, necessitates the control of contamination. The issue of contamination issue has been flagged as a big concern for tissue sample analysis as compared to blood (34). Irreproducibility has also been a concern previously with regards to the element concentrations in the colon tissues (35). The authors had also pointed out the interference of structural composition variation of the tissues with tumor transformation (35).

Here, we highlight several important elements associated with human health and cancer. From our review, CRC patients generally showed an increased level of Cobalt (Co) and Sulphur (S) in their serum. Co is an important component in vitamin B12 and essential for human wellbeing (20). A higher concentration of Co in the circulation is thought to be primarily due to occupational exposure (36) but none of the studies had assessed this parameter. Moreover, co-induced carcinogenicity has been demonstrated in cell lines and experimental animal models by causing DNA breaks and inhibition of DNA repair (17). A higher concentration of S in the CRC serum and tissue samples is postulated as a consequence of impaired S element metabolism due to hypoxia in tumor environment. This compromised condition may alter the redox activity and its ability to stick to specific molecules (37) thus leading to the abundance.

Zn is a vital element playing major roles in DNA synthesis, gene expression, various enzymes activities and maintenance of normal human growth (38). As part of functional superoxide dismutase (SOD), Zn delivers the anti-oxidative effect and protects against carcinogenesis by removing free radicals and activating the DNA repair mechanism (17). Previous supplementation studies have suggested the protective role of Zn against carcinogens in several gastrointestinal cancers, including CRC (39–41).

Selenium (Se) is a key element in several seleno-proteins that are essential for good health such as glutathione peroxidase (GPx) (38) In the form of selenoproteins, Se plays an important role in structural and enzymatic functions and is best-known as an antioxidant. Low or diminishing concentrations of Se are associated with the development of cancer (42–45). Investigation on CRC using an animal model showed clear suppression of apoptosis and inflammation-mediated carcinogenesis by GPx2 (46). Inversely, adequate Se supply has been associated with better protection against inflammation, apoptosis, and carcinogenesis in a clinical trial study (47).

Cu is the main building block for more than 30 enzymes in the human body including ceruloplasmin, ascorbate oxidase, lysine oxidase, dopamine-hydroxylase, cytochrome oxidase and tyrosinase (20). Mobilization and redox activity of catalytic Cu have been suggested to play key roles in the production of reactive oxygen species (ROS) (48). Binding of Cu to DNA bases in the chromatin could damage the DNA strands and is thought to be a key initiating step of carcinogenesis (49, 50).

There were no significant changes reported for Boron (B) and Ca from our extracted data probably due to unadjusted values in relation to different environmental exposure such as smoking and diet (51). Previous studies have shown some evidence that relates these elements strongly to cancer. Boron has been used as anticancer agents (52) while a Swedish AMORIS cohort study proved that changes in Ca level may confer a higher risk to CRC (53).

Mg is another essential element in our body responsible for absorption and utilization of nutrients such as carbohydrates, fatty tissues, and proteins. Mg acts as a cofactor for many enzymes in the metabolic pathways and as a direct antagonist of intracellular calcium (38). High intracellular levels of Mg^2+^ has been reported to confer a metabolic advantage to the neoplastic cells in addition to causing the alteration in the genome (54) and cell proliferation (55).

A strong connection between Fe and cancer has been well documented. Over-secretion of lipocalin 2, an iron-binding protein will lead to increased iron content in the adipose tissues, thus will trigger the adverse effect of iron overload and consequently oxidative damage which leads to CRC development (38). A cohort study in Taiwan has reported a J-shaped curve between serum Fe and cancer risk, with higher cancer risk for individuals with Fe concentration below 60 mg/dL or above 120 mg/dL (56). Additionally, the free irons which are not bound to any ligand could also affect negatively on human health (57). Manganese (Mn) is present at approximately ten milligrams in the human body, mostly in the bone, liver and kidney (58). Mn serves as a cofactor for several important enzymes including arginase, cholinesterase, mitochondrial superoxide dismutase and several phosphatases, phosphoglucomutase, pyruvate carboxylase, peptidases and glycosyltransferases (59). A low level of Mn was strongly associated with breast cancer (60). The mitochondrial manganese superoxide dismutase (MnSOD) is known to inhibit cell growth in different tumor cell lines (61). Overexpression of MnSOD has been reported to slow down the growth of HCT116 human colorectal cancer cells by inducing cellular senescence (62).

The carcinogenic mechanism of action for Plum-bum (Pb), also known as lead, is presumed to be via the interference with the DNA repair processes (17). Pb-induced oxidative stress includes damage to the cell membrane, DNA, key enzymes such as catalase, SOD, GPx, and glucose-6-phosphate dehydrogenase (G6PD), and the pool of non-enzymatic antioxidant molecules such as thiols (63).

Animal and plant food products are the main sources of cadmium (Cd) in our body (20). The carcinogenic activity of Cd was first discovered in animals and subsequently in humans (64). Previous data had suggested the possible mechanism of action of Cd via indirect processes or epigenetic changes that suppresses apoptosis or activates the oncogenes (65).

Methyl bromide is a component of Bromine (Br) which is highly effective as a fumigant and is widely used in pesticides. The epidemiological and toxicological evidence had suggested a potential relationship between the exposure of methyl bromide and severe human health problems, including cancer (66). However, the carcinogenic mechanism remains unclear in spite of its presence in ubiquitous but trace amount in our body. Last but not least is Chromium (Cr), an important element in energy metabolism. In cells, Cr(VI) which is the most carcinogenic form of Cr, are metabolized by several mechanisms to its reduced species along with ROS generation. The process by which Cr may induce neoplastic progression has been implied as both complex and elusive with evidence suggesting its role in the formation of DNA-protein cross-linkages (67).

### Future direction of trace elements study and CRC

Our systematic review has revealed the limited data and lack of standardized assessment on environmental exposures (demographic, lifestyle and diet) linked to the levels of trace elements in our body. This inadequacy contributes to the inconsistent results which may be due to unadjusted values with different levels of exposures. Evidence has implied the influence of these exposures onto the concentration of trace elements. Different levels of physical activity (68) weight category (38, 69), smoking status (70), alcohol consumption (71) and diet (72, 73) were reported to also influence the concentrations. Therefore, there is a need to look more specifically into environmental exposures, which are likely to disclose changes in the level of the elements. Not only that the assessment of exposures is crucial, but the different ages within the study population (74), gender (75) or existence of comorbidity (76) may also interact exogenously with these elements.

The majority of the previous studies focused more on metal-induced carcinogenicity and lacked the approach of a multi-element assessment. Research on heavy metals has generally established the linkage to cancer either at higher or lower concentrations. There is still a need to study other elements as they may be the potential biomarkers for early detection of cancer or further developed as chemopreventive agents. However, many phases of discovery research and validation studies must be conducted before any cutoff values can be established. The need to investigate the role of these trace elements in a cohort setting cannot be disregarded in order to classify them as a definite risk factor and not just an association. With the advancement of technology in modern instruments with greater sensitivity, specificity and high-throughput capability such as the ICP-MS will certainly enable researchers to do more screening and group profile studies.

Likewise, the interaction between the trace elements also needs to be explored further. Oxidative stress, antioxidants and trace element levels should be measured simultaneously to provide further insight into their central roles particularly in the mechanisms of carcinogenesis. This can be scientifically addressed by conducting in vitro and in vivo studies. Genomic research may also provide clues on how genetic polymorphisms and mutations may impact on the trace elements and their biological functions.

## Conclusion

Differential levels of trace elements were demonstrated in both serum and tissue samples of CRC patients as compared to healthy controls. Both deficiency and excess of trace elements are potentially harmful to patients and may be related to CRC development. In this systematic review, limited databases were searched due to institutional’s limited access, therefore the exploration may be incomprehensive.

## Ethical considerations

Ethical issues (Including plagiarism, informed consent, misconduct, data fabrication and/or falsification, double publication and/or submission, redundancy, etc.) have been completely observed by the authors.
